# Metagenome-assembled genome of the alkaliphilic* Cyanobacterium* sp. PNNL-SSL1

**DOI:** 10.1128/mra.01398-25

**Published:** 2026-03-19

**Authors:** Hossein Khadivar, Huyen Bui, Michael Huesemann, Song Gao, Robin Gerlach

**Affiliations:** 1 Center for Biofilm Engineering, Montana State University-Bozeman 33052 , Bozeman, Montana, USA; 2 Department of Chemical and Biological Engineering, Montana State University-Bozeman 33052 , Bozeman, Montana, USA; 3 Marine and Coastal Research Laboratory, Pacific Northwest National Laboratory 497252 , Sequim, Washington, USA; Rochester Institute of Technology, Rochester, New York, USA

**Keywords:** alkaliphilic cyanobacterium, bioproducts, microalgae, biofuels, high pH

## Abstract

Microalgae and cyanobacteria are promising sources of fuels, chemicals, and bioproducts, but CO_2_ supply increases production cost. We present the metagenome-assembled genome of the alkaliphilic *Cyanobacterium* sp. strain PNNL-SSL1 obtained from Soap Lake (Washington, USA). PNNL-SSL1 shows strong potential for biomass production using only atmospheric CO_2_, reducing cultivation expenses.

## ANNOUNCEMENT

Biomass from microalgae including cyanobacteria is a potential alternative to petroleum (1–3). A limitation for growing microalgae in large scales is supplying sufficient CO_2_ in concentrated form, which can result in high production and delivery cost (2, 3). Alkaliphilic microalgae have been investigated because high amounts of inorganic carbon can be provided through bicarbonate–carbonate buffered systems (2–5). Here, we present a linear metagenome-assembled genome (MAG) of an alkaliphilic cyanobacterium, *Cyanobacterium* sp. strain PNNL-SSL1, obtained from a naturally alkaline ecosystem: Soap Lake, WA, USA (47°23′36″ N, 119°29′05″ W), which has pH ~10 and ~100 mmol/L inorganic carbon concentrations (6). PNNL-SSL1 has been shown to be promising for biomass production, relying solely on atmospherically derived CO_2_ (3).

The PNNL-SSL1 MAG was obtained by sequencing the metagenomes of two xenic cultures dominated by PNNL-SSL1 named SSL1-A and SSL1-B. These xenic cultures were maintained in NCMA Spirulina medium (7) at ~22°C under ~450 µmol/m^2^/s with 12 hour light:12 hour dark light cycle and diluted weekly to sustain active growth. A cell pellet from each culture (~150 µL) was resuspended in 150 µL of 2× DNA/RNA Shield buffer (Zymo Research, USA) and stored at –80°C. DNA was extracted with the ZymoBIOMICS DNA Kit (Zymo Research), quantified using a NanoDrop spectrophotometer (Thermo Scientific), and sent to the Joint Genome Institute (JGI). At JGI, libraries were prepared with insert sizes of 200–300 bps using KAPA Biosystems HyperPrep library kit (Roche) and sequenced on an Illumina NovaSeq S4 platform to obtain 150-bp paired-end reads (331,537,726 reads for SSL1-A; 528,729,554 reads for SSL1-B). Sequencing reads downloaded from the JGI genome portal were filtered and assembled into contigs as a co-assembly using MEGAHIT (v1.2.9) (8). Contigs were binned using the KBase apps CONCOCT (v1.1) and MetaBAT2 (v1.7) and optimized with DAS-Tool (v1.1.2) (9–11). The Anvi’o platform (v7.1) was used for manual bin curation, as well as estimation of completeness and redundancy based on copy numbers of single-copy core genes in each bin (12). Binned contigs were assembled into MAGs and annotated with PROKKA (v1.14.5) (13), and genus-level relative abundances were calculated based on coverage in Anvi’o. Taxonomy was determined using the KBase app GTDB-Tk (v2.3.2) (14). Default parameters of software packages were used.

The co-assembled metagenome contained 19,177 linear contigs ≥1 kb, which were grouped into 52 bins using MetaBAT2 based on the nucleotide content and coverage. The DAS-tool optimization resulted in 29 bins. The only cyanobacterial MAG obtained in the co-assembled metagenome was estimated to be 97.18% complete with 1.41% redundancy. Features of the resulting genome and its annotation are listed in Table 1. The MAG was identified to belong to the genus *Cyanobacterium*, which is consistent with the primary classification of SSL1 by PNNL (3). The closest placement taxonomy, *Cyanobacterium* sp002813895, was determined by GTDB-Tk based on the Genome Taxonomy Database (14). Realignment of sequencing reads to the co-assembled metagenome (Bowtie v2.3.2 in KBase) showed that 328,724,316 reads were aligned to the cyanobacterial MAG, equivalent of ~16,042× coverage (15).

**TABLE 1:** Genome assembly statistics for the metagenome-assembled genome of *Cyanobacterium* sp. strain PNNL-SSL1

PNNL-SSL1 key statistics	Values
Genome size (bp)	3,073,714
Number of contigs	69
GC content (%)	38.38
N50 (bp)	82,559
Number of CDS	2,779
Number of tRNAs	38
Number of tmRNAs	1
Number of reads	328,724,316
Read length (bp)	150
Completeness (%)	97.18

## Data Availability

The raw data for SSL1-A (SRX29282892) and SSL1-B (SRX29282893) are available in the National Center for Biotechnology Information (NCBI) Sequence Read Archive (SRA) database under BioProject accession number PRJNA1280515. The obtained co-assembly was deposited on Dryad; https://datadryad.org/dataset/doi:10.5061/dryad.31zcrjf28. *Cyanobacterium* sp. PNNL-SSL1’s BioSample is deposited under the accession number SAMN50708828. This Whole Genome Shotgun project has been deposited at DDBJ/ENA/GenBank under the accession JBSZCP000000000. The version described in this paper is version JBSZCP000000000.1. The individual metagenome assemblies of SSL1-A (aka SSL1-WT culture) and SSL1-B (aka SSL1-Turbo culture) were originally deposited in the Department of Energy Joint Genome Institute Integrated Microbial (IMG) Genomes database under IMG 291693 and IMG 291694, respectively.
